# Camera fusion for real-time temperature monitoring of neonates using deep learning

**DOI:** 10.1007/s11517-022-02561-9

**Published:** 2022-05-03

**Authors:** Simon Lyra, Jöran Rixen, Konrad Heimann, Srinivasa Karthik, Jayaraj Joseph, Kumutha Jayaraman, Thorsten Orlikowsky, Mohanasankar Sivaprakasam, Steffen Leonhardt, Christoph Hoog Antink

**Affiliations:** 1 Medical Information Technology, Helmholtz Institute for Biomedical Engineering, RWTH Aachen University, Pauwelsstr. 20, 52074 Aachen, Germany; 2 Section of Neonatology, RWTH Aachen University, 52074 Aachen, Germany; 3 Department of Electrical Engineering, Indian Institute of Technology, 600036 Madras, Chennai, Tamil Nadu, India; 4 Saveetha Medical College, 602105 Kanchipuram, Saveetha, Nagar, Chennai, India; 5 KIS*MED (AI Systems in Medicine), Electrical Engineering and Information Technology, TU Darmstadt, 64283 Darmstadt, Germany

**Keywords:** Camera fusion, Deep learning, Infrared thermography, Neonatal intensive care unit

## Abstract

**Abstract:**

The continuous monitoring of vital signs is a crucial aspect of medical care in neonatal intensive care units. Since cable-based sensors pose a potential risk for the immature skin of preterm infants, unobtrusive monitoring techniques using camera systems are increasingly investigated. The combination of deep learning–based algorithms and camera modalities such as RGB and infrared thermography can improve the development of cable-free methods for the extraction of vital parameters. In this study, a real-time approach for local extraction of temperatures on the body surface of neonates using a multi-modal clinical dataset was implemented. Therefore, a trained deep learning–based keypoint detector was used for body landmark prediction in RGB. Image registration was conducted to transfer the RGB points to the corresponding thermographic recordings. These landmarks were used to extract the body surface temperature in various regions to determine the central-peripheral temperature difference. A validation of the keypoint detector showed a mean average precision of 0.82. The registration resulted in mean absolute errors of 16.4 px (8.2 mm) for *x* and 22.4 px (11.2 mm) for *y*. The evaluation of the temperature extraction revealed a mean absolute error of 0.55 $^{\circ }$C. A final performance of 31 fps was observed on the NVIDIA Jetson Xavier NX module, which proves real-time capability on an embedded GPU system. As a result, the approach can perform real-time temperature extraction on a low-cost GPU module.

**Graphical abstract:**

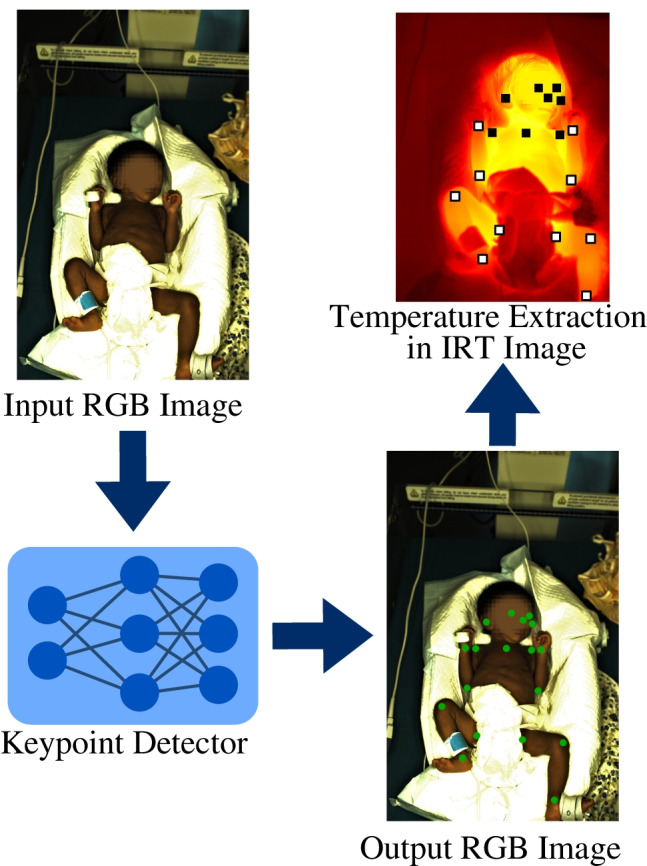

## Introduction

Prematurity is the leading cause of death for children under the age of 5 years and shows increasing rates on a global scale. From the 15 million premature infants born every year, approx. one million children die due to various complications of preterm birth [1], although three-quarters of these deaths could be prevented by proper neonatal care [2]. On a neonatal intensive care unit (NICU), immature patients receive medical care in an incubator, which offers a protected environment regarding temperature, humidity, and oxygen concentration. Furthermore, the health condition of the infant is monitored by measuring key vital signs such as heart rate (HR), respiration rate, temperature, and oxygen saturation. Therefore, clinical surveillance systems enable the diagnosis of complications, which can be a result of an immature organ system: Neonates, especially if born preterm, are highly susceptible to infections. As a result, infection remains one of the leading causes of mortality and morbidity in early human life. The clinical picture in postnatal neonatal sepsis presents as systemic inflammatory response syndrome (SIRS), with a consecutive shock and rapid deterioration of ventilation, circulation, and metabolism [3]. Although a crucial early detection of sepsis remains a challenge due to a subtle disease progression, infection-typical symptoms such as a central-peripheral temperature difference (cpTD) offer potential to support the diagnosis [4].

Today, patient monitoring is conducted using adhesive sensors, which require direct contact with the patient’s skin. Although the cable-based vital sign measurement is a crucial aspect of neonatal care, the attached electrocardiogram electrodes, temperature probes, and the pulse oximeter cuff can cause discomfort and stress for the infant. They can also lead to side effects such as medical adhesive-related skin injuries, which result in wounds and infections [5]. To overcome these disadvantages and potential vital risks for the development of neonates, camera-based techniques provide an approach for unobtrusive monitoring. While sensitive CMOS cameras can be used to monitor heart rate [6] and respiration [7], an infrared thermography (IRT) device can be applied to measure the surface temperature distribution of a patient [8, 9]. Furthermore, the combination of several CMOS cameras and IRT devices (camera fusion) increases the number of simultaneously monitored vital signs and could improve the quality of specific signals due to redundant measurements.

The automatic and robust camera-based measurements of vital signs require advanced algorithms to extract regions in an image, which can be used for unobtrusive monitoring. However, classical approaches for head detection or body part segmentation in multi-modal datasets reach their limits due to great computational effort and strong dependencies on the recording conditions. Over the past years, the progress in the fields of deep learning (DL) and GPU computing facilitated the application of high-performing models, which are robust and real-time feasible. However, due to the lack of public neonatal image datasets, the availability of pre-trained models is crucial for algorithmic development. Furthermore, the fine-tuning of pre-trained approaches (transfer-learning) for multi-modal data can potentially improve camera-based monitoring by applying the results of a prediction from one modality to another. For instance, the availability of RGB datasets and pre-trained models for detection or segmentation is much bigger than for the IRT modality, so the thermographic analysis could be improved by applying the RGB results to IRT images. This could be used for improved measurement of temperature distribution in thermograms, using an RGB-based DL model to predict locations on the skin and transform them into the IRT domain. Despite their potential, those approaches require image registration for proper analysis. Nevertheless, such techniques have not yet been covered in the literature on neonatal unobtrusive monitoring.

In this paper, a DL-based approach for temperature monitoring of neonates in a NICU using a multi-camera system is presented. A transfer-learned keypoint detector was used for the prediction of body landmarks in an RGB dataset, which was recorded in a clinical environment. After an image registration step, the transformation of the RGB landmarks to the IRT frames was performed, so the body surface temperature was extracted in regions-of-interest (ROIs) around the detected body points. Subsequently, the cpTD on the body surface was computed. In a final step, a performance analysis was conducted to analyze the real-time capability of the algorithm on embedded GPU modules.

## Related works

Since the very first camera-based measurement of dermal perfusion changes in 2000 [10], different modalities have been evaluated by many research groups [11]. Besides a large range of mono-modal approaches in the literature, the amount of studies in the field of camera fusion for neonatal monitoring has increased recently. In 2014, Cattani et al. used a multi-camera setup and maximum-likelihood detection for seizure prediction in neonatal patients [12]. While Lorato et al. used three low-cost IRT devices to improve the camera-based respiration extraction in 2020 [13], Paul et al. combined several (near infrared) monochrome and RGB cameras to evaluate the signal quality for monitoring the neonatal pulse rate [14]. In 2021, Lorato et al. published a multi-modal approach for respiration monitoring in infants using a combination of thermal and RGB videos [15]. Since camera fusion uses multi-modal data and therefore requires image registration, several approaches were investigated to relate RGB and IRT images. Here, the mutual information (MI) criterion (see Section 4.2) proved to be a suitable metric of image matching to implement a registration method. Although MI is widely used for image registration of e.g. radiological data, it can be sensitive to outliers, which are visible in one of the two images. It also suffers from local and biased maxima [16]. While several techniques for robust MI-based image registration were published for intensity nonuniformity [17] and for time-shifted MRI recordings [18], the incidence of unmatchable outliers was expected to be low for the neonatal RGB/IRT dataset, because time-synchronous images with similar viewing angles were registered.

In recent years, novel DL-based approaches showed potential to improve camera-based vital sign measurement. In 2019, Villarroel et al. published a DL-based algorithm to automatically segment skin areas of an infant and estimate vital signs only when the patient was present in the field of view [19]. Furthermore, in 2019, Chaichulee et al. used convolutional neural networks (CNNs) for cardio-respiratory signal extraction from RGB video recordings [20]. In the same year, Ornek et al. applied a CNN to IRT data to detect the health status of neonates [21]. In 2020, Navaneeth et al. published an approach to classify respiratory diseases in infants using a DL model in thermal imaging [22] and in 2021, Ervural et al. applied CNNs for a classification of neonatal diseases in thermograms [23]. Recently, Khanam et al. published an article where the DL-based object detector YOLOv3 was applied for the extraction of heart rate and respiration [24].

While the described literature was focused on neonatal applications, further research was recently conducted in the field of ROI tracking and signal fusion for vital signs extraction for adult data. In 2019, Pursche et al. analyzed the improvement of signal quality of camera-based HR monitoring from human faces using CNN-based ROI tracking [25]. In 2021, Kurihara et al. published a method for non-contact HR estimation using adaptive fusion of RGB and near-infrared images based on the analysis of background illumination variations [26]. Furthermore, Liu et al. proposed a multi-modal quasi-contactless sensor based on RGB images and a ballistocardiogram for HR measurement under extreme facial poses and large-motion disturbances using a novel landmark-based approach for a facial ROI [27].

Next to DL-based object detectors and computationally complex segmentation algorithms, the extraction of body landmarks using keypoint detection approaches is becoming more popular due to high accuracy and performance. These detectors are used to locate body parts and joints and therefore can support the extraction of ROIs for unobtrusive vital sign measurements. In 2017, Cao et al. introduced CMU-Pose for real-time multi-person 2D body pose estimation (BPE) [28], which was further developed and published as the already well-known OpenPose algorithm in 2019 [29]. The features are analyzed by a multi-stage CNN to generate confidence maps (CMaps) and part affinity fields (PAFs) in RGB images. A CMap is a 2D representation of the likelihood that a particular body part can be located in any given pixel. A PAF is a set of 2D vector fields, which encodes the degree of association between body parts of different people in an image [28]. In contrast to top-down approaches, which first detect a person and then detect the keypoints, Cao et al. used a bottom-up approach to find keypoints first to form the person skeleton. Therefore, the detection performance is independent of the number of people present in the image.

Since OpenPose was released, research groups have applied it to RGB images for neonatal motion analysis [31, 32] and posture detection [33]. In this work, the keypoint detector was used in combination with multi-modal images for temperature monitoring of neonates.

## Materials and methods

### Experimental setup and dataset

The multi-modal dataset for this study was recorded in the NICU of Saveetha Medical College and Hospital, Chennai, India, while the trials were approved by the institutional ethics committee of Saveetha University (SMC/IEC/2018/03/067). Written informed consent was obtained from the parents of all patients. In total, the study included 19 stable patients with gestational ages at birth between 29 and 40 weeks. The ages varied from 37 h to 56 days post-birth and their weights ranged from 1500 to 3010 g. All infants were recorded with a multi-camera setup, which was equipped with the IRT camera VarioCAM HD head 820 S (InfraTec, Germany), an RGB camera of type Grasshopper 3 GS3-U3-23S6C-C (FLIR, USA), and a monochrome camera of type Grasshopper 3 GS3-U3-23S6M-C (FLIR, USA). The monochrome device was equipped with a green interference filter for unobtrusive heart rate measurement. However, these recordings will not be used in this work and are only mentioned for completeness. The temperature measurements were conducted with 1,024 $\times$ 768 IR pixels (16-bit), a temporal resolution of 10 Hz, and a thermal sensitivity of 20 mK. Furthermore, the RGB and MONO cameras recorded at 60 Hz with a resolution of 1,920 $\times$ 1,200 pixels (12-bit) using fixed focal length lenses of type Fujinon CF12.5HA-1 (Fujifilm, Japan). All cameras were attached to a 3-mm-thin aluminum base plate. Four OLED panels of type Keuka warm white (OLEDWorks, Germany) were mounted on the base plate using self-designed 3D-printed acrylonitrile-butadiene-styrol (ABS) frames — printed with a Prusa i3 MK3S (Prusa, Czech Republic) — for a patient-friendly illumination from several directions. The advantages of using OLEDs instead of LEDs were described in [34]. The recordings were conducted in a dark environment using only OLED light illumination.

**Fig. 1:**
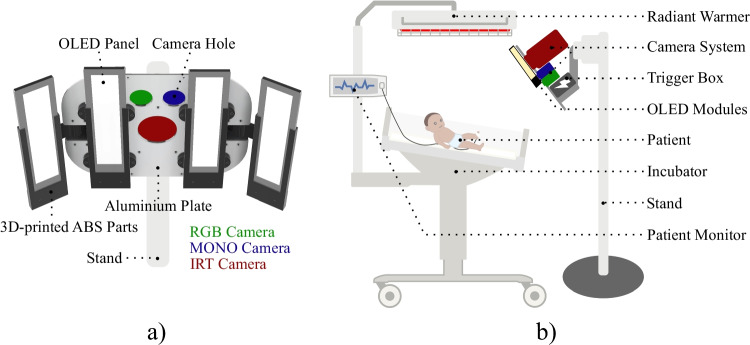
**a** Patient view of the camera setup modified from [34]. **b** Recording setup at a radiant warmer in the NICU

As illustrated in Fig. 1a, the cameras were arranged in a triangular formation. The setup was attached to a stable stand, which was positioned in a window-free room. The neonates were placed next to the setup in an open incubator with an attached radiant warmer. A Radical-7 pulse oximeter (Masimo, USA) was used to record reference data for HR and oxygen saturation. The infants were recorded for a length of 10 min. The measurement setup is depicted in Fig. 1b.

Eighteen of all 19 patients were in a supine position during the measurements. Since one patient was in a prone position and removed from the incubator several times for medical care, the data of this specific neonate was excluded from training and validation. A dataset was created from all RGB recordings by randomly sampling 150 frames from every patient to train and validate the DL-based keypoint detector. As shown in Table 1, this resulted in a dataset of 2,700 RGB images (DL dataset).

**Table 1:** Dataset sampling for training and evaluation

Usage	Modality	Patients	Images	Total
DL dataset	RGB	18	150	2,700
cpTD dataset	RGB	18	20	360
	IRT	18	20	360

**Fig. 2:**
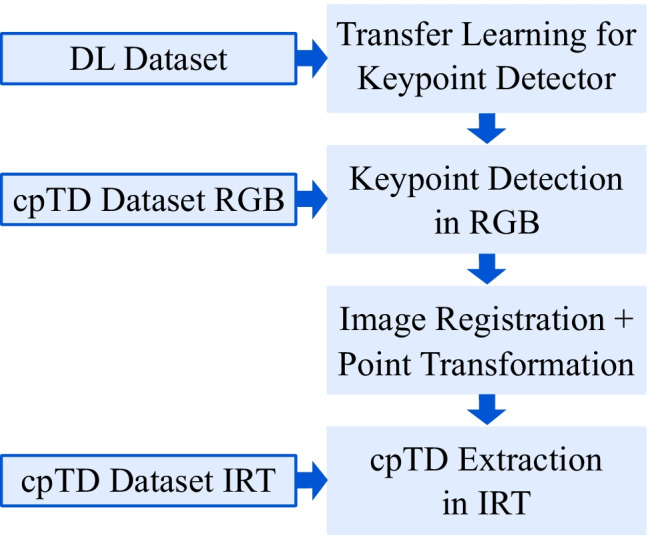
Overview of the dataset usage for the DL-based approach

In Fig. 2, an overview of the DL-based algorithm and the use of the described datasets are presented. The RGB-trained keypoint detector should be applied to IRT recordings for temperature extraction. Therefore, an additional subset of data was created termed cpTD dataset. It consists of 20 randomly selected RGB frames and 20 corresponding IRT frames per subject.

### Data preprocessing

For the transfer learning step, a pre-trained keypoint detector was adapted to the neonatal RGB dataset. Therefore, the sampled images from the DL dataset (see Table 1) needed to be labeled with body landmarks: the ground truth (GT) keypoint labeling was performed using the tool “COCO Annotator” [35], which is a web-based image annotation tool from Brooks et al. to export annotations in the well-known Microsoft Common Objects in Context (COCO) format [36]. In each frame, the following 17 person keypoints were labeled (if available): nose, left eye, right eye, left ear, right ear, left shoulder, right shoulder, left elbow, right elbow, left wrist, right wrist, left hip, right hip, left knee, right knee, left ankle, right ankle.

**Fig. 3:**
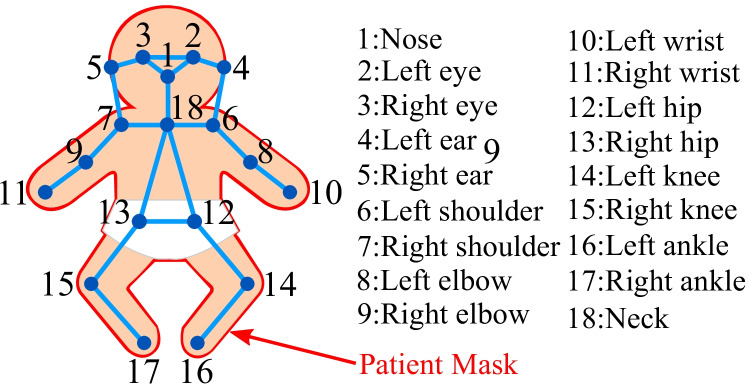
Annotation example with 18 keypoints and connections

Since the evaluation step of COCO datasets requires GT keypoints and the area of the detected subject, a patient mask was annotated in the frames. A labeled example with the patient mask, all keypoints, and corresponding point connections, which show the body pose of the patient, is illustrated in Fig. 3.

As the approach used in this work further predicted the neck location, this keypoint was added using the center of both shoulder points. In addition to the labeling of the RGB frames for training and validation of the DL approach, the IRT dataset and the corresponding RGB images (see cpTD Dataset in Table 1) were annotated for performance analysis of the later conducted keypoint transformation and measurement of GT body surface temperature.

### Keypoint detection

In this work, the NVIDIA AI IOT project trt_pose was used, which enables a real-time BPE in RGB images on embedded GPUs in Python by applying the NVIDIA tool TensorRT (NVIDIA, USA) [37]. With TensorRT, trained neural networks can be optimized to maximize performance for inference deployment. The trt_pose architecture is based on the model structures of both CMU-Pose and OpenPose and was optimized by NVIDIA for real-time applications.

In contrast to CMU-Pose (see Section 2), trt_pose adapted the model architecture. Since Xiao et al. proposed residual neural networks (ResNets, [38]) as a superior network architecture to extract image features for pose estimation [39], a ResNet-18 backbone instead of the initial VGG-19 layers was used. Furthermore, the architecture was adapted from NVIDIA according to the OpenPose model: instead of using a two-branch multi-stage CNN where the stages in both branches were interconnected, the interdependencies were removed and both the CMap and PAF branches were adapted. Therefore, deconvolutional layers were added in both branches as upsampling stages to generate high-resolution feature maps as proposed in [39]. Subsequently, convolutional layers generate the keypoints and connections of body parts, which were passed into a greedy algorithm written in C++ to receive the body pose. The associations between localized body parts and the assignment of these parts to a unique person are performed using a bipartite graph. This leads to a NP-hard graph matching problem, which is solved using the Munkres (Hungarian) algorithm [40]. Therefore, the edges in the graph (body points) are first weighted by computing the line integral along the vector connecting two nodes to evaluate the PAF output tensor of the model. Finally, the total score of the graph is maximized, which results in the human pose.

## Implementation

### Detector training and validation

Since the size and variability of the neonatal dataset were not sufficient to train a robust model for BPE from scratch, transfer learning was conducted using provided model weights from trt_pose, which were pre-trained on the COCO 2017 keypoint dataset (including over 150,000 people and 1.7 million labeled keypoints in RGB images) [36]. For the transfer learning step, a high-performance desktop computer was used, running Ubuntu 18.04 and featuring an Intel Xeon E5-2620 processor, an NVIDIA Quadro RTX5000, and 64 GB RAM. The GPU was deployed in combination with CUDA 11.0, cuDNN 8.0.5, OpenCV 4.4.0, and Torchvision 0.8.1 in Python 3. The training process can be described as follows: the ColorJitter functionality from Torchvision was used as a data augmentation tool that randomly changed the brightness, contrast, saturation, and hue of an image. Due to the invariable measurement conditions and the resulting low variability in recording perspective and distance, it was assumed that the training process would not benefit from additional, classical augmentation methods, such as rotation, flipping, or scaling, which was already observed in previous publications [41]. Thus, no additional augmentation strategies were investigated.

A patient-wise 18-fold leave-one-out cross-validation (LOOCV) was performed to measure the keypoint detection performance during the training steps. In contrast to a patient-wise cross-validation (CV), where one fold contains several patients in the training and in the test set, the patient-wise LOOCV is a configuration in which the data of only one patient forms the test set while the remaining data is used for the training process. Although the computational costs of a LOOCV can be higher compared to a CV, it was preferred since the neonatal dataset was relatively small compared to the COCO dataset, which was used to pre-train the detection model. Therefore, overfitting as a challenge for small datasets and related biased estimates of model performance were addressed. However, the test errors in LOOCVs can have a higher variance as only one fold is used for prediction [42].

The training was conducted during a maximum of 250 epochs on a high-performance GPU, whereby the learning rate was reduced after 75 and 100 epochs from the initial value of 0.001 by a factor of ten. Furthermore, early stopping was used to prevent overfitting by evaluating the loss during the training process. After the training, the model was optimized using TensorRT (see Section 3.3) and the COCO evaluation tool was used to analyze the performance of the keypoint detector. The average precision (AP) and average recall (AR) were computed in analogy to the intersection over union (IoU) in the validation of object detectors. The IoU serves as a similarity metric between GT and prediction to specify if a prediction is correct using a threshold for overlap, which allows calculating AP and AR. In analogy, the object keypoint similarity (OKS) was defined in [43] as a metric for keypoint detection. It takes euclidean distances between each corresponding GT and detected keypoint, a visibility flag, a per-keypoint constant, and the object scale into account. By thresholding the OKS to define a prediction as correct or incorrect, AP and AR can be computed. While AP uses the mean AP over 10 OKS thresholds (.50:.05:.95), the metric $\mathrm {AP}^{75}$ describes the AP for the threshold of 0.75.

### Image registration

Since the body points were predicted in the RGB frames, an image registration needed to be done to transform the RGB keypoints to the thermographic recordings for temperature measurement. Although both cameras recorded from the same angle and distance to the neonates, the translational displacement and the optical distortion due to the different camera lenses required a registration to map the body points from RGB to IRT. In general, image registration is the process of transforming different datasets into one coordinate system.

As the multi-modal images had different properties for feature extraction (e.g., colorful objects could be invisible in IRT images due to their temperature), which complicated the feature matching, basic concepts to extract a projective transformation (homography) such as the findHomography functionality from OpenCV failed. Therefore, a custom approach for intensity based image registration was implemented, which is based on the mutual information (MI) criterion. This metric is a measure of image matching that allows the signal to be different in both images. It measures the prediction quality of the signal in the second image, given just the signal intensity in the first, using the joint (2D) histograms. The MI of two variables is defined by

1 $$\begin{aligned} I(X,Y) = \sum \limits _{x,y} p(x,y)\,\log \left( \frac{p(x,y)}{p(x)p(y)}\right) , \end{aligned}$$

where *p*(*x*) and *p*(*y*) in Eq. 1 are the marginal probability mass functions and *p*(*x*, *y*) is the joint probability mass function [44]. A high MI corresponds to a good match of the images. However, as described in Section 2, MI can be sensitive to outliers visible in one image. Because the images were recorded simultaneously, time-shifted outliers were not expected in the neonatal dataset.

**Fig. 4:**
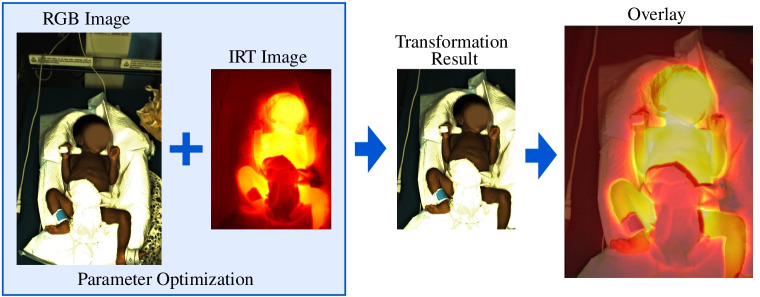
Overview of the image registration algorithm. The final overlay image was resized for illustration

Since an affine transformation step was necessary for the registration, an optimization problem to maximize the MI was defined to derive the entries of the matrix. The optimization was solved using the Python tool hyperopt [45]. Furthermore, two crop variables for *x* and *y* directions were added as parameters, because a high MI could only be achieved by matching images with the same field of view. As illustrated in Fig. 4, the optimization used both the RGB and IRT images as input and finds the optimal crop in the RGB image and the transformation matrix by maximizing the MI. As a result, the transformed RGB image and the IRT frame can be overlaid. The optimization step was conducted during 500 epochs. The transformation matrix and the crop parameters were used to register the RGB and IRT images. Furthermore, the RGB points could be transformed into the IRT frame.

Although the measurement setup had a fixed position for all recordings, the distance between the camera system and the subjects varied among the neonates. This can be explained by the fact that the infants were carried in an open incubator from the patient room to the measurement setup, whereby the exact same position could not be restored between the measurements. Furthermore, the position of the patient in the incubator varied. Thus, a registration per patient was necessary.

### Vital signs extraction

**Fig. 5:**
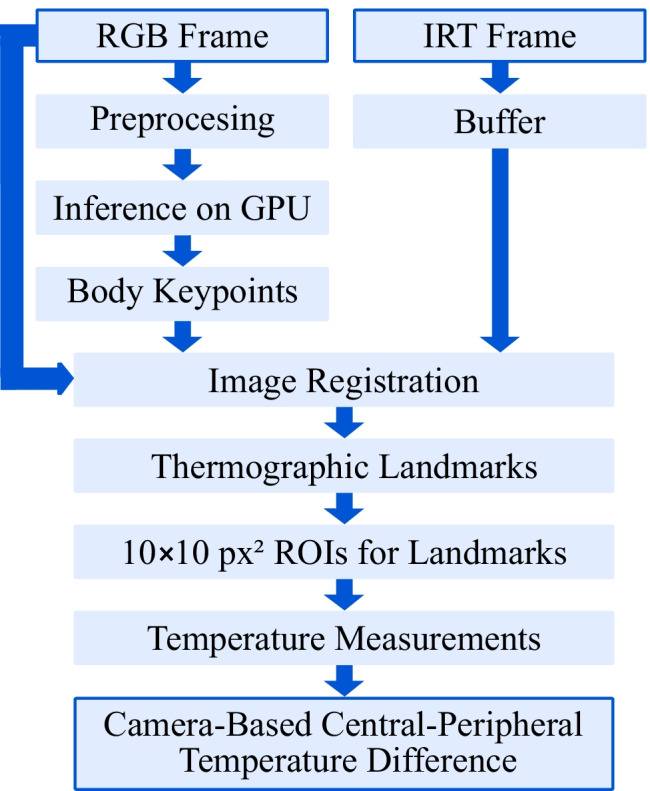
Overview of the algorithm for temperature measurement

The proposed approach uses camera fusion to automatically detect the cpTD on the body surface of neonates from IRT data by applying the results of a BPE in RGB to thermograms. An overview of the algorithm is presented in Fig. 5.

**Fig. 6:**
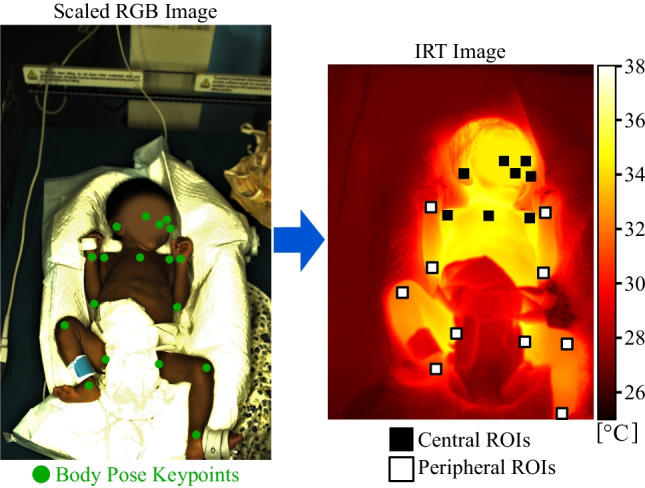
Application of the proposed algorithm to an image pair. The RGB image and the ROIs in the IRT frame were rescaled for illustration purposes only

In an initial step, the RGB and IRT frames were loaded. While the RGB frame was preprocessed for the detection model (rescaling and GPU upload), the IRT frame was stored in a buffer. Subsequently, the inference step was conducted on the GPU, which resulted in the body keypoints. To exclude a training effect during prediction and enable a proper evaluation of the algorithm, the model weights from a LOOCV fold were used, in which the particular patient was not in the training dataset. The RGB keypoints were then transformed using the affine transformation matrix described in Section 4.2. The resulting IRT points determined the ROI center locations for the following temperature measurement. As depicted in Fig. 6, the body points from RGB detection were classified as central or peripheral in the IRT frame. While facial points and landmarks in the upper body region were marked as central, the remaining keypoints formed the peripheral group. In the next step, all available ROIs in the IRT frame were used to determine the maximum temperature value in the region. A size of $10\times 10$ $\mathrm {px^2}$ was used for the ROIs. Finally, a mean temperature was computed for central and peripheral regions to compute the cpTD on the skin surface of the neonates.

### Real-time feasibility on embedded GPUs

To show the feasibility of real-time performance of the trained detector, the inference time of the algorithm on different (embedded) GPU systems was analyzed. The NVIDIA low-cost system-on-modules Jetson Xavier NX (approx. 400$, 08/21) and the higher performant Jetson AGX Xavier (approx. 700$, 08/21) (NVIDIA, USA) were used for inference. Both development boards provide a 64-bit CPU and use a NVIDIA Volta GPU, including tensor cores and 16 GB (AGX Xavier) and 8 GB (Xavier NX) of RAM. Since these modules provide high performant GPUs on a low-cost single chip, they can be used for the implementation of efficient embedded systems for real-time camera-based monitoring [46]. The performance analysis is presented in Section 5.1.

## Results

### Keypoint detection performance

The results of a patient-wise 18-fold LOOCV for the keypoint detection algorithm in RGB images are presented in Table 2. For every fold, the data of one patient was excluded from the training process and used for validation.

**Table 2:** Results of a leave-one-out cross validation

Fold (patient)	AP	$\mathrm {AP}^{75}$	AR
1	86.6	98.0	90.5
2	76.2	98.0	80.7
3	88.3	99.0	90.4
4	75.5	97.9	77.0
5	69.1	84.7	72.9
6	88.5	100.0	91.3
7	93.5	98.0	94.4
8	57.4	61.7	57.8
9	98.7	100.0	99.0
10	76.2	84.4	79.3
11	36.0	18.9	38.6
12	96.5	100.0	97.9
13	99.4	100.0	99.6
14	93.0	100.0	94.7
15	87.9	99.0	91.8
16	71.7	71.2	74.3
17	98.9	100.0	99.2
18	79.5	95.8	84.7
Mean	81.8	89.3	84.1
SD	16.4	20.7	16.0
CMU-Pose [28]	69.3	67.5	−
OpenPose [29]	70.7	71.3	−
PRTR [47]	73.3	79.9	80.2

Additionally, the detection results for CMU-Pose, OpenPose, and PRTR (a state-of-the-art regression-based approach, which uses cascade transformers [47]) on the COCO 2017 keypoint validation set were provided to classify the results in the context of keypoint detection. In general, the results show a mean AP of 81.8 and mean $\mathrm {AP^{75}}$ of 93.2. The mean AR was obtained as 84.1. While for some patients very high values for AP and AR were observed, e.g., fold 13 (AP: 99.4, $\mathrm {AP}^{75}$: 100, AR: 99.6), the results for folds 8 and 11 in contrast show only weak results for the keypoint detection. This can be explained by highly divergent patient-specific characteristics in these folds (see Section 6.1). As expected for a LOOCV, this resulted in corresponding high values for the standard deviation (SD) (AP: 16.4, AR: 16.0). The varying APs and ARs also indicated that overfitting was prevented during the training process.

In comparison to the results of CMU-Pose, OpenPose, and PRTR, which were trained and evaluated using the COCO keypoint dataset, the proposed transfer learning process showed superior results. These outcomes were expected because the neonatal recordings were reduced in size and variability compared to the COCO dataset.

The inference process of keypoint detection was conducted on embedded GPU systems. In Table 3, the performance for keypoint inference and the total algorithm on different GPU modules were presented in frames per second. As expected, the highest performance was achieved using the NVIDIA Quadro RTX 5000 (98 fps in total). Nevertheless, both Jetson modules also showed real-time feasibility for the algorithm. While for the Xavier NX, a total performance of 31 fps was obtained, the AGX Xavier achieved 47 fps. A comparison of the total computation time with the inference performance revealed that the keypoint detection was conducted much faster than the entire algorithm. This can be explained by the CPU-based pre-processing of the images and post-processing for temperature measurement in the body ROIs (see Fig. 6). Due to the (NumPy) mean operations for up to 36 $10\times 10\,\mathrm {px}^{2}$ ROIs with float temperature values per image, the total performance was depreciated. However, real-time execution on low-cost GPU modules was achieved.

**Table 3:** Mean performance on different GPU platforms

Platform	Inference (fps)	Total (fps)
Jetson Xavier NX	98	31
Jetson AGX Xavier	165	47
Quadro RTX 5000	560	98

### Image registration performance

As mentioned in Section 4.3, the validation of the image registration to transform the RGB keypoints to thermographic frames was conducted for every fold of the patient-wise LOOCV. The results of the patient-wise process are presented in Table 4. The labeled IRT points were used as GT and compared to the transformed points. The mean absolute error (MAE) and the SD for every fold and the corresponding total error metrics for the *x* and *y* components in pixels can be obtained.

**Table 4:** Results of the point transformation in pixels and millimeters

	$\mathrm {MAE}_{\mathrm {X}}$(px)		$\mathrm {MAE}_{\mathrm {Y}}$(px)		$\mathrm {MAE}_{\mathrm {X}}$(mm)		$\mathrm {MAE}_{\mathrm {Y}}$(mm)	
Fold	Mean	SD	Mean	SD	Mean	SD	Mean	SD
1	4.7	1.6	20.5	2.6	2.4	0.8	10.3	1.3
2	11.2	2.4	43.3	4.7	5.6	1.2	21.7	2.4
3	14.8	7.3	9.7	5.6	7.4	3.7	4.9	2.8
4	12.3	2.6	11.6	2.9	6.2	1.3	5.8	1.5
5	20.9	7.1	46.2	6.7	10.5	3.6	23.1	3.4
6	14.4	3.2	11.4	4.1	7.2	1.6	5.7	2.1
7	37.4	5.1	58.1	9.8	18.7	2.6	29.1	4.9
8	9.6	1.0	15.6	0.9	4.8	0.5	7.8	0.5
9	16.7	3.8	12.5	6.1	8.4	1.9	6.3	3.1
10	15.5	9.9	15.3	8.1	7.8	5.0	7.7	4.1
11	16.7	12.9	15.5	9.3	8.4	6.5	7.8	4.7
12	20.3	2.1	11.8	2.2	10.2	1.1	5.9	1.1
13	28.9	3.1	28.5	3.1	14.5	1.6	14.3	1.6
14	16.0	10.3	18.1	5.8	8.0	5.2	9.1	2.9
15	12.3	1.0	7.3	0.6	6.2	0.5	3.7	0.3
16	8.5	0.6	8.2	0.7	4.3	0.3	4.1	0.4
17	13.7	7.7	31.8	10.2	6.9	3.9	15.9	5.1
18	16.4	9.4	22.4	15.6	8.2	4.7	11.2	7.8
Total	16.4	9.4	22.4	15.6	8.2	4.7	11.2	7.8

While high accuracies with MAEs and SDs, less than 10 px, were achieved for several folds in *x* direction (e.g., folds 1, 8, 16), the total metrics for $\mathrm {MAE_X}$ and $\mathrm {MAE_Y}$ revealed errors of 16.4 px respectively 22.4 px. Furthermore, the total SDs were determined as 9.4 px for *x* and 15.6 px for *y*. Since a distance between the camera setup and the subjects of approx. 1 m results in a spatial resolution of 0.5 mm per px, the mean errors are in the range of 10 mm. In general, the analysis of the total evaluation results showed higher errors for *y* components in comparison to the *x* values, which were associated with the orientation of the IRT camera and the position of the patient in the incubator (see Section 6.1).

**Fig. 7:**
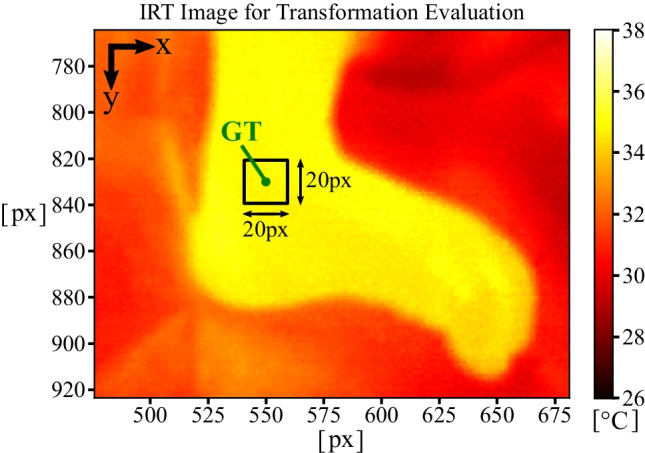
Illustration for classification of pixel errors for point transformation

For a better classification of the obtained registration errors, Fig. 7 illustrates a crop from a thermogram with a visible neonatal foot. In the infrared frame, the GT point for the left ankle was labeled, and a $20\times 20\,\mathrm {px}^{2}$ ROI was highlighted to show a potential error of 20 px, which was in the range of the mean MAE for both directions. Since the registration process revealed maximum errors of up to 37.4 px in *x* and 58.1 px in *y* direction for some folds, the outcomes showed that for outliers a region outside the highlighted ROI in Fig. 7 could be extracted. However, the mean results showed that the transformed points were still found on the surface of the extremities in the IRT frames. Therefore, the registration can be used for temperature extraction. Since for every extracted $20\times 20\,\mathrm {px}^{2}$ ROI the maximum value was extracted for temperature measurement on the body surface (see Section 4.3), deviations in the range of 20 px in both *x* and *y* directions should still enable a monitoring of local temperatures due to a sufficient pixel coverage.

### Temperature measurements

**Fig. 8:**
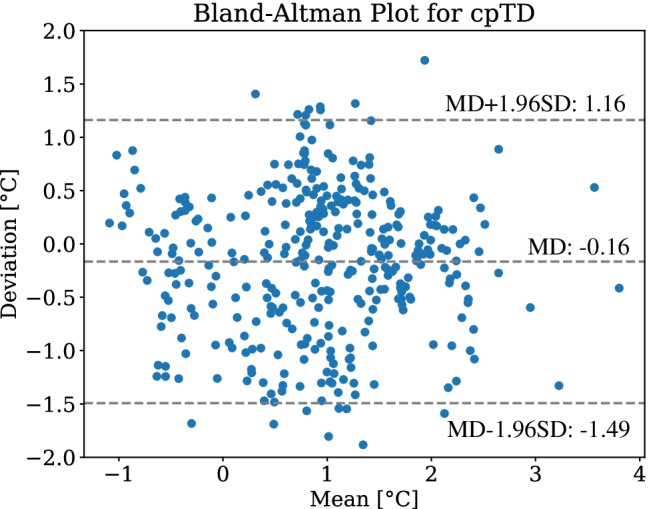
Bland-Altman plots for extraction of cpTD

The transformed RGB keypoints were used for extraction of maximum temperatures in the ROIs to determine the cpTD from the thermographic images. The cpTD was determined for every frame of a fold and compared with the GT using the predicted and labeled keypoints in the thermogram. Subsequently, the mean value and the SD were computed for all frames of each subject. In Table 5, the averaged MAEs and the corresponding SDs for every fold and additionally the total metrics for all images are presented. The analysis of the Bland-Altman plot in Fig. 8 revealed outliers and further showed a large proportion of negative deviations from the GT cpTD. While in a Bland-Altman plot the *y*-axis shows the deviation between GT and the actual extracted value, the *x*-axis represents the mean value of both values.

**Table 5:** Results of the temperature extraction

	MAE cpTD ($^{\circ }$C)	
Fold	Mean	SD
1	0.77	0.32
2	0.73	0.76
3	0.37	0.41
4	0.47	0.62
5	0.51	0.42
6	0.67	0.47
7	0.97	0.38
8	0.24	0.12
9	0.40	0.49
10	0.45	0.68
11	0.35	0.44
12	0.53	0.62
13	0.45	0.46
14	0.88	0.56
15	0.21	0.22
16	0.51	0.61
17	0.36	0.37
18	0.98	0.58
Total	0.55	0.67

The results showed a total MAE of $0.55\,^{\circ }$C for the cpTD with a SD of $0.67\,^{\circ }$C. While for fold 15 a minimum MAE of $0.21\,^{\circ }$C was obtained, the maximum error was determined to be $0.98\,^{\circ }$C. The Bland-Altman plot in Fig. 8 displays slightly negative mean differences (MD). Thus, the proposed algorithm underestimated the GT cpTD. This effect can be explained by inaccurate keypoint predictions, so the defined ROIs contained lower maximum values for temperature extraction.

Furthermore, negative mean values were noticeable, which correspond to a negative (and therefore unphysiological) cpTD. Due to different measurement conditions regarding the clothing of the neonates, the cpTD could result in a negative value, if central or peripheral body parts were covered. In the conducted study, the clothing of the subjects was not predefined in any way. Still, for all subjects, central and peripheral measurement locations could be extracted due to exposed hands or feet. The issue of only a few found keypoints e.g., due to medical care during the measurements, further distorted the extraction (see Section 6.2). However, all predicted keypoints were used for temperature analysis to demonstrate the feasibility of local temperature monitoring.

## Discussion

### Keypoint detection and image registration

**Fig. 9:**
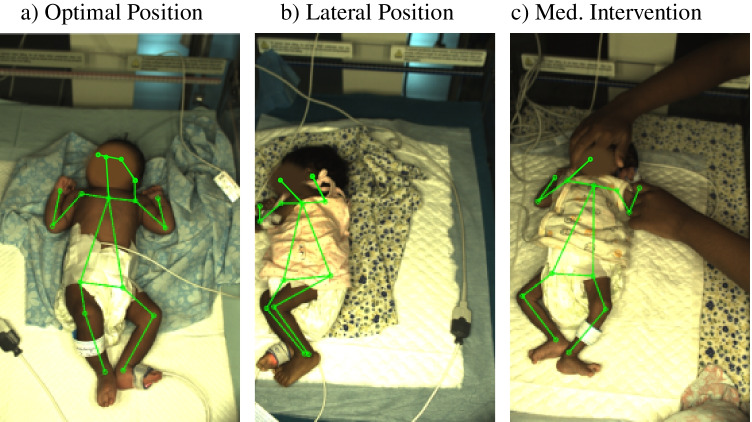
Different levels of distortions during measurements: **a** no distortion, **b** strong lateral position leads to less detected points, and **c** medical intervention results in occlusions and corrupted detections

The results of the LOOCV for the DL-based keypoint detection in general showed a higher value for $\mathrm {AP}^{75}$ in comparison to the AP. The fixed threshold of 0.75 resulted in a more frequent occurrence of very high or low (see fold 11) values of $\mathrm {AP}^{75}$. This is due to the fact that a higher/lower $\mathrm {AP}^{75}$ is obtained more often, since only the majority of keypoints must have an OKS above/below 0.75 regardless of the exact value. In contrast, the AP smoothed these effects by considering 10 different OKS thresholds. Since the validation was performed for every fold, patient-specific variations become visible directly in the evaluation results. The folds 8 and 11 showed only weak results for the keypoint detection. These detection results can be explained by a higher frequency of medical interventions, which resulted in occlusions and detection of false positives. For illustration, optimal measurement conditions and two different levels of distortions are presented in Fig. 9. For lateral positions and medical interventions, the number of faulty predictions was increased.

Next to the promising results for several folds, the analysis of the registration process, where the RGB points were transformed to the IRT images, showed a mean MAE of 22.4 px (11.2 mm) for the *y* direction, which can be explained by the IRT camera orientation and the varying absolute position of the patient in the incubator. Since the device was rotated to adapt the field of view for the measurements (wide sensor side oriented to the longitudinal axis of the subject), the optical distortion was higher compared to the *x* direction (transversal axis) of the sensor. However, the position of the patient in the incubator during the measurement has proven to be very important, too. For infants with less movement and a central position in the incubator close to the camera, the optical distortions in *y* direction (longitudinal axis) were less, which resulted in minor errors. Furthermore, the errors in both *x* and *y *directions were increased for patients who moved to the image borders. Especially the accuracy for the point detection of limbs, which were close to the image borders, was decreased due to the effect of increasing distortion effects towards the edges. However, the results revealed that the point transformation from RGB to IRT images was sufficiently accurate to measure the temperatures on the body surface locally.

While in Fig. 9a optimal recording conditions can be observed, Fig. 9b shows limbs outside the frame and a lateral position of the subject, which led to less detected points due to a more difficult detection task. Furthermore, in Fig. 9c, occlusions caused by medical intervention are visible. Although the transfer learning step was conducted, in some frames, the limb keypoints of the medical staff were still found, which resulted in faulty detections. For these situations, keypoints could get lost, resulting in a lower number of evaluable locations. The weakest detection results for fold 11 result from a medical indication (legs in plaster cast), which complicated the detection of the lower limbs. Since the model from this fold was not trained for patients with such a medical condition, the prediction was distorted.

Despite the described limitations, the evaluation of the transfer learning step showed very promising results for the application of pre-trained models on a neonatal dataset. In future work, the model accuracy could be further improved by using more labeled data of patients with specific medical conditions in the training step. Nevertheless, the prediction results and the real-time capability of the algorithm on low-cost embedded GPUs showed a potential implementation of a continuous measurement system. To achieve a real-time system, no computationally expensive segmentation algorithms were used, but the concept of a higher performant keypoint detector was applied for the extraction of body regions.

### Temperature extraction

The temperature extraction showed promising results for a DL-based monitoring of the cpTD. Since a mean MAE of $0.55^{\circ }$ was achieved, the feasibility of an automated system for clinical surveillance of the cpTD to, e.g., monitor sepsis-related temperature progressions was demonstrated. However, the algorithm still showed several limitations: in the event of a medical intervention, occlusions and wrong predicted keypoints complicated the detection of body landmarks, which was forwarded to the temperature extraction. Furthermore, the cpTD monitoring was distorted due to body regions outside the frame for some subjects. Since the DL detector even found occluded keypoints due to overlapping body parts, the extraction could be corrupted in an event of interleaved central and peripheral regions. Due to these limitations and the fact that clothing was not specified during the study in order not to interfere with daily clinical care, negative cpTD were observed.

In future work, the algorithm could be enhanced by an analysis to derive the number of actual skin pixels in extracted ROIs. Due to differences in color between covered body parts and skin, a method based on the RGB pixels could be implemented for clothing detection. Furthermore, keypoints detected on limbs of medical staff during interventions could be neglected by excluding limb keypoints that were assigned to a person with no central keypoints. Since the DL approach allows counting the detected persons, only keypoints that are connected to a minimum number of predicted central points could be used for analysis.

Since no reference data was measured for cpTD during the camera recording, the presented results could not be validated with gold-standard GT temperature. However, the focus of this work was set into the measurement of relative differences in skin temperature using an IRT device. Nevertheless, in future work, the inclusion of a GT cpTD, e.g., from incubator skin sensors, could support the implementation of an automated alarm system for a camera-based early detection of a disease progression that is related to a cpTD in real time.

## Conclusion

In this paper, we presented a DL-based approach for the real-time extraction of local surface temperatures from a multi-modal clinical dataset of neonates. An RGB dataset of 18 subjects was used for a transfer-learning step of a pre-trained body keypoint detector. A LOOCV revealed promising results of 81.8% for the AP, which indicates a robust prediction of body landmarks. The patient-wise image registration was conducted using a computed transformation matrix. The evaluation of the transformed points showed total MAEs of 16.4 px (8.2 mm) for *x* and 22.4 px (11.2 mm) for *y* direction. The temperature extraction was performed in the thermogram by evaluating ROIs, which were located using the transformed body points. During a performance evaluation on embedded GPUs, real-time capability of 31 fps was observed using the low-cost module Jetson Xavier NX, which indicated a highly efficient algorithm.

Although the proposed technique showed promising results for the DL-based cpTD extraction, the analysis of the results revealed several challenges. Next to lower prediction accuracies for patients with medical indications, the point transformation showed a patient-dependent distortion, which changed with the position of the subject in the image. Furthermore, since a patient-wise matrix calculation was required for the transformation, an application of the proposed algorithm in a clinical scenario would be dependent on an initial calibration process to conduct the registration step. However, these problems will be addressed in future work by using more data for transfer learning of the keypoint detector and a more advanced DL-based method for image registration, such as described in [48]. The prediction step will be further developed by adding a temporal keypoint tracker, so the complete loss of landmarks during motion into e.g. a lateral position will be reduced. Here, previously detected keypoints and the level and direction of the movement will be used. Additionally, more clinical data with adequate GT skin temperature needs to be processed with the algorithm to prove the applicability of robust bedside monitoring in a NICU.

In conclusion, the overall results showed the feasibility for the use of low-cost embedded GPUs for real-time temperature monitoring of neonates. Simultaneously, the keypoint detection further enables a movement tracking to quantify the state of activity of the patient. Although only a comparable small dataset was used for transfer learning, promising outcomes were obtained, which encourages other research groups with datasets of similar size to use DL-based techniques. The novel real-time cpTD extraction from multimodal recordings revealed the advantages of camera fusion and promotes the development of smart devices for the camera-based early detection of clinical symptoms for different pathologies such as neonatal sepsis. Thus, this work represents a step towards the replacement of potentially harmful cable-based sensors in neonatology.
